# Efficacy and Safety of Acute Phase Intensive Electrical Muscle Stimulation in Frail Older Patients with Acute Heart Failure: Results from the ACTIVE-EMS Trial

**DOI:** 10.3390/jcdd9040099

**Published:** 2022-03-27

**Authors:** Shinya Tanaka, Kentaro Kamiya, Yuya Matsue, Ryusuke Yonezawa, Hiroshi Saito, Nobuaki Hamazaki, Ryota Matsuzawa, Kohei Nozaki, Masashi Yamashita, Kazuki Wakaume, Yoshiko Endo, Emi Maekawa, Minako Yamaoka-Tojo, Takaaki Shiono, Takayuki Inomata, Junya Ako

**Affiliations:** 1 Department of Rehabilitation, Nagoya University Hospital, Nagoya 466-8560, Japan; s-tanaka@med.nagoya-u.ac.jp; 2 Department of Rehabilitation, School of Allied Health Sciences, Kitasato University, Yokohama 252-0373, Japan; myamaoka@med.kitasato-u.ac.jp; 3 Department of Rehabilitation Sciences, Graduate School of Medical Sciences, Kitasato University, Yokohama 252-0373, Japan; mss.yamashita.pt@gmail.com; 4 Department of Cardiovascular Biology and Medicine, Juntendo University Graduate School of Medicine, Tokyo 113-8421, Japan; yuya8950@gmail.com (Y.M.); saito.hiroshi@kameda.jp (H.S.); 5 Department of Rehabilitation, Kitasato University Medical Center, Saitama 364-8501, Japan; yonekko@insti.kitasato-u.ac.jp (R.Y.); wakaume@insti.kitasato-u.ac.jp (K.W.); 6 Department of Rehabilitation, Kameda Medical Center, Chiba 296-8602, Japan; endo.yoshiko@kameda.jp; 7 Department of Rehabilitation, Kitasato University Hospital, Yokohama 252-0375, Japan; hamanobu0317@gmail.com (N.H.); 0818.n.kohei@gmail.com (K.N.); 8 Department of Physical Therapy, School of Rehabilitation, Hyogo University of Health Sciences, Kobe 650-8530, Japan; ryota122560@gmail.com; 9 Research Fellow of Japan Society for the Promotion of Science, Tokyo 102-0083, Japan; 10 Department of Cardiovascular Medicine, Kitasato University School of Medicine, Yokohama 252-0374, Japan; emimae1207@med.kitasato-u.ac.jp (E.M.); jako@kitasato-u.ac.jp (J.A.); 11 Department of Cardiovascular Medicine, Kitasato University Medical Center, Saitama 364-8501, Japan; shiono@med.kitasato-u.ac.jp; 12 Department of Cardiovascular Medicine, Niigata University School of Medical and Dental Sciences, Niigata 951-8510, Japan; inotaka@med.niigata-u.ac.jp

**Keywords:** electrical muscle stimulation, frail, physical function, muscle strength, acute decompensated heart failure, exercise, early rehabilitation

## Abstract

As frailty in older patients with acute heart failure (AHF) has an adverse effect on clinical outcomes, the addition of electrical muscle stimulation (EMS) to exercise-based early rehabilitation may improve the effects of treatment. Post hoc analysis was performed on a randomized controlled study for clinical outcomes and prespecified subgroups (ACTIVE-EMS: UMIN000019551). In this trial, 31 AHF patients aged ≥ 75 years with frailty (Short Physical Performance Battery [SPPB] score 4–9) were randomized 1:1 to receive treatment with an early rehabilitation program only (*n* = 16) or early rehabilitation with add-on EMS therapy (*n* = 15) for 2 weeks. Changes in physical function and cognitive function between baseline and after two weeks of treatment were assessed. There were no adverse events during the EMS period. The EMS group showed significantly greater changes in quadriceps’ isometric strength and SPPB compared to the control group, and EMS therapy showed uniform effects in the prespecified subgroups. There were no significant differences in the changes in other indexes of physical function and cognitive function between groups. There was no significant difference in the rate of heart failure hospitalization at 90 days between groups. In conclusion, older AHF patients with frailty showed greater improvement in lower extremity function with the addition of EMS therapy to early rehabilitation without adverse events.

## 1. Introduction

Frailty, defined as reduced physiological reserve and vulnerability to stressors, is a high-priority issue in cardiovascular medicine in older patients [1,2]. Acute heart failure (AHF) is a leading cause of hospitalization, and is associated with frailty, reduced quality of life (QOL), and increased medical costs [3]. In older AHF patients, frailty is a strong predictor of early disability, readmission, and mortality [4].

Significant improvements in physical function and QOL, as well as reduced risk of hospitalization, have been reported in patients with heart failure (HF) undergoing exercise-based cardiac rehabilitation [5,6]. However, there have been few reports regarding the effects of rehabilitation programs in older patients with frailty hospitalized for HF. In addition to the symptoms of HF, frail older HF patients show hemodynamic instability, including dyspnea and fatigue, and exercise intolerance, which adversely affect their ability to participate in exercise therapy. Therefore, it is necessary to develop novel interventions to prevent early injury and enhance functional improvements in frail older patients with AHF. Electrical muscle stimulation (EMS) is a method of safely inducing muscle contraction that does not require volitional effort on the part of the patient and does not evoke dyspnea. EMS can be applied easily, is well tolerated, and has been shown to be safe for use in HF patients, including older patients and those with severe disease status [7]. Several months of EMS therapy in patients with HF has been shown to have a number of beneficial effects on muscle strength, exercise capacity, and health-related QOL [8]. Short-term EMS was shown to improve muscle strength, exercise capacity, and health-related QOL in hospitalized HF patients; these studies included few older patients [9,10].

We recently showed favorable short-term effects of acute phase intensive EMS on physical function in frail older patients with AHF (ACTIVE-EMS trial) [11]. The ACTIVE-EMS trial was designed to address a number of high-priority gaps in evidence of the efficacy of exercise-based cardiac rehabilitation programs in frail older patients with AHF. Previous clinical trials excluded recently hospitalized HF patients and included few older patients [5,6]. The recent large-scale REHAB-HF trial showed that the initiation of multidomain rehabilitation therapy in the hospital and its continuation following discharge had beneficial effects, leading to improvements in physical function at three months in frail older patients with HF [12]. Except in severe cases, participation in outpatient cardiac rehabilitation programs was shown to be associated with reduced risks of all-cause mortality and hospital readmission due to HF in frail patients with HF [13]. However, as the rate of implementation of outpatient cardiac rehabilitation is very low (<10%), only a small proportion of patients can benefit from such interventions [14,15]. Older patients with AHF are frequently frail and have both severe and widespread impairments in physical function on hospital admission [16], and hospital-acquired disability was shown to be associated with increased risk of all-cause mortality and hospital readmission due to HF [17]. Although acute-phase initiation of cardiac rehabilitation has been reported to be associated with better short-term clinical outcomes in patients hospitalized for AHF [18], it is often difficult for frail older patients with AHF to participate in exercise-based therapy due to dyspnea, fatigue, exhaustion, and exercise intolerance. ACTIVE-EMS is a clinically important trial suggesting that the usefulness of EMS, which can be initiated in hospital during the period of bed rest and inactivity when the majority of muscle reduction occurs, is an adjunct or a bridge to rehabilitation in frail older patients with AHF. To understand the efficacy and safety profiles of EMS in this high-risk population, we set out to examine the safety of EMS and its effects on physical function in prespecified subgroups for the ACTIVE-EMS trial, and to evaluate the prognostic significance of short-term EMS therapy.

## 2. Materials and Methods

### 2.1. Trial Design

The ACTIVE-EMS trial was a multicenter, randomized, prospective, 2-week, open-label, controlled, superiority trial with two parallel groups. The study design and primary results have been described elsewhere [11,19]. Two university hospitals and one public hospital in three prefectures in Japan participated in this trial. The trial was conducted in accordance with the principles of the Declaration of Helsinki and the institutional review board/ethics committee at each participating hospital provided approval. The trial was registered at the University Hospital Medical Information Network Clinical Trials Registry before enrollment of the first patient (Identifier: UMIN000019551). The trial was performed in accordance with the Consolidated Standards of Reporting Trials (CONSORT) Extended Non-Drug guidelines [20].

### 2.2. Patients and Randomization

The target population consisted of older patients aged ≥ 75 years admitted to the participating hospitals due to acute exacerbation of HF within 5 days, and with frailty defined as functional limitations of the lower extremities assessed using the Short Physical Performance Battery (SPPB, score 4–9) when they became hemodynamically stable [21]. Patients requiring assistance with walking 1 month before hospitalization, those with a diagnosis of acute coronary syndrome on admission, and those with severe symptomatic aortic stenosis, mitral stenosis, or hypertrophic obstructive cardiomyopathy were excluded. Full details of the inclusion and exclusion criteria are provided in Table S1. Baseline measurements were made in eligible patients who provided written informed consent to participate in the trial. The patients were then randomly assigned in a 1:1 ratio to receive early rehabilitation with add-on EMS therapy (EMS group) or early rehabilitation only (control group) using a centralized, web-based system, with block randomization, which was stratified according to clinical site. The allocation results were disclosed to the participants and investigators, including those involved in assessment of the outcomes.

### 2.3. EMS Protocol

**Figure 1:**
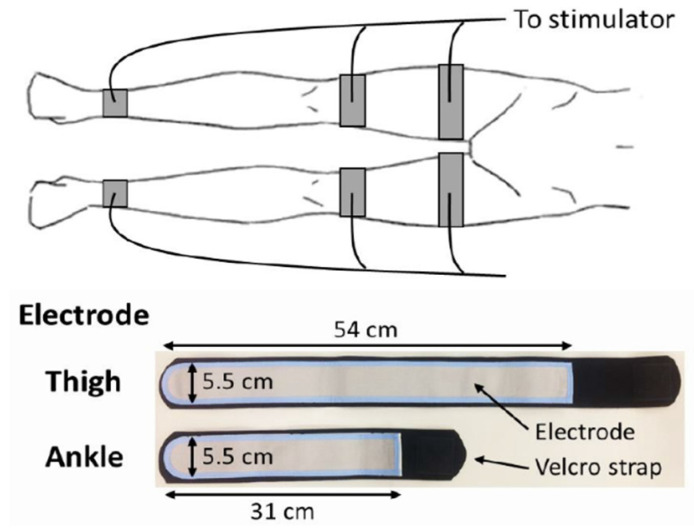
Belt electrode skeletal muscle electrical stimulation.

A belt electrode skeletal muscle electrical stimulation system (Auto Tens Pro; Homer Ion Co., Ltd., Tokyo, Japan) was used for application of EMS to all muscle groups of both legs, including the quadriceps femoris, hamstrings, tibialis anterior, and triceps surae muscles [22,23]. Six silicon–rubber electrode bands were applied to the proximal and distal parts of the thigh and distal parts of the lower leg and held in place with Velcro straps (Homer Ion Co., Ltd., Tokyo, Japan) (Figure 1). The stimulator current waveform produced co-contractions in the muscle groups of the lower extremities at a frequency of 20 Hz with a pulse width of 250 μs, and a duty cycle consisting of stimulation for 5 s with a pause for 2 s [22,24]. Both legs were stimulated simultaneously as the stimulation cycles were synchronized for the bilateral legs. Visible muscle contractions were induced by setting the output intensity to the maximum tolerable value without discomfort or pain. Trained physiotherapists who were members of the investigation team applied EMS for 30–40 min per day, 5 days per week, for up to 2 weeks.

### 2.4. Early Rehabilitation Program

In this study, a multi-domain rehabilitation intervention involving supervised rehabilitation therapy with two exercise stages was applied by a multi-disciplinary team to improve strength, balance, mobility, and endurance, utilizing reproducible, targeted exercises in elderly HF patients. The first stage of the supervised rehabilitation therapy program consisted of gradual mobilization, which included basic activity training, such as sitting up in bed, sit-to-stand motion, and walking within the hospital ward. The patients proceeded to the second stage once they were shown to be clinically stable, which consisted of a gym-based exercise training program with 5 min of stretching, balance training, and resistance training using the patient’s own body weight, and 20–40 min of aerobic training using a cycling ergometer or treadmill walking, including warm-up and cool-down periods. The exercise intensity in both types of training was prescribed at a rating of perceived exertion (RPE) of 11–13 on the Borg RPE scale (range: 6–20). The exercise intensity was increased progressively in each session. Patients participated in the rehabilitation program for 1 h daily, 5 days per week, during the period of hospitalization, as long as there were no adverse symptoms or events.

### 2.5. Outcomes

The change in quadriceps isometric strength (QIS) between baseline and 2 weeks was set as the primary endpoint of the ACTIVE-EMS trial, because it has been shown to be a good predictor of both exercise capacity and mortality [25,26], and frail older AHF patients show impaired strength of muscles in the lower extremities [16]. The highest values of QIS on the right and left sides were averaged and expressed as a percentage relative to body weight (%BW). The changes in physical function based on handgrip strength, SPPB score, usual gait speed, and 6-min walking distance (6MWD) and cognitive function assessed using the Digit Symbol Substitution Test (DSST) between baseline and 2 weeks were determined as secondary endpoints. Clinical events during the intervention period, including the incidences of acute kidney injury, worsening of renal function, and worsening of HF, and changes in B-type natriuretic peptide level between baseline and 2 weeks were evaluated as secondary outcomes to determine the clinical safety and feasibility of EMS therapy. The outcomes were measured at hospital discharge in patients discharged before the end of the intervention period. The rates of all-cause mortality and hospital readmission due to HF within 90 days were also assessed.

The Mini-Cog instrument was administered at baseline to screen for the presence or absence of cognitive impairment. The MOS 36-Item Short-Form Health Survey physical functioning scale, Frailty score, and SARC-F questionnaire were also administered at baseline to measure frailty and sarcopenia. A triaxial accelerometer (TH-400; Yamasa Corp., Tokyo, Japan) was used to measure physical activity throughout the 2-week study period. The outcome assessors from each participating hospital were trained to use a standardized method and were provided with a manual that included photographs of the outcome assessment method to minimize interobserver variation.

### 2.6. Sample Size Calculation

The required sample size for between-group analyses of the primary outcome measure of the ACTIVE-EMS trial was calculated using G*Power 3 analysis set for F-test analysis of covariance [27]. We anticipated that Cohen’s f effect size would be 0.40 (large) for the primary outcome based on previous studies [28]. The calculations indicated that a total of 52 patients (26 patients per group) would be required for a power of 80% and an alpha value of 5%. Taking a dropout rate of 30% and rounding into account, we planned to enroll 80 patients in this study [19].

### 2.7. Statistical Analysis

Normally distributed continuous variables are presented as the mean ± standard deviation (SD), non-normally distributed variables are presented as the median and interquartile range (IQR), and categorical variables are shown as counts and percentages. Analyses were performed on an intention-to-treat basis. The unpaired t test or Mann–Whitney U test was used for continuous variables, and Fisher’s exact test was used for dichotomous variables. To examine the potential consistency of the effects of the intervention on the primary outcome, subgroup analysis was also performed using forest plots in the following prespecified subgroups: age (stratified at 85 years), sex, left ventricular ejection fraction (LVEF, stratified at 50%), baseline QIS (stratified at the median value), and baseline 6MWD (stratified at the median value) [19]. Subgroup analyses were also performed for all secondary outcomes that showed a significant intervention effect. The relationships of change in QIS with changes in body weight, BNP level, and other functional variable were evaluated using Spearman’s rank correlation.

Statistical analyses were performed with SPSS version 28.0 (IBM Corp., Armonk, NY, USA) and R version 3.2.1 (R Foundation for Statistical Computing, Vienna, Austria). In all analyses, a two-tailed *p* < 0.05 was taken to indicate statistical significance.

## 3. Results

**Figure 2:**
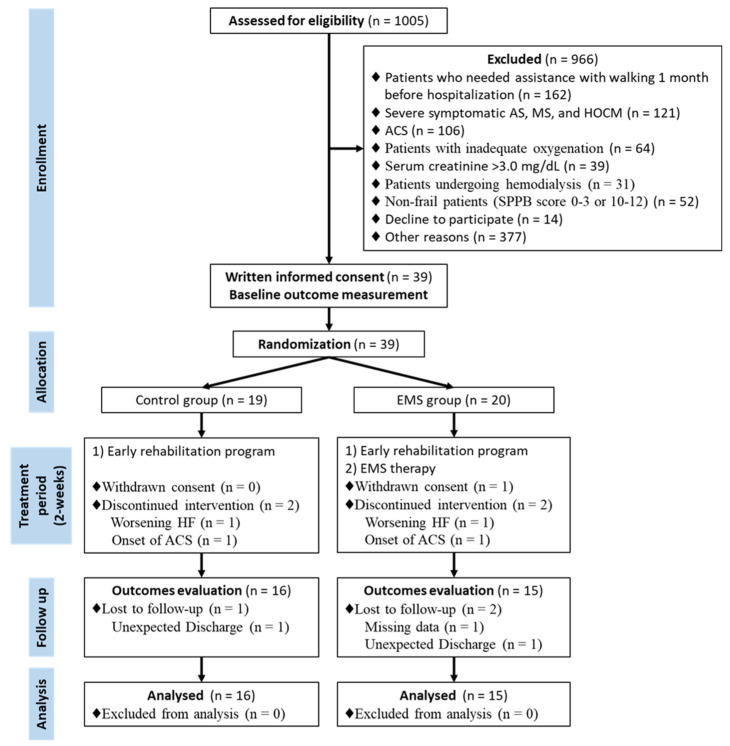
Study flow diagram. ACS, acute coronary syndrome; AS, aortic stenosis; EMS, electrical muscle stimulation; HF, heart failure; HOCM, hypertrophic obstructive cardiomyopathy; MS, mitral stenosis; SPPB, short physical performance battery.

The ACTIVE-EMS trial enrolled its first and last patients in June 2016 and March 2019, and 31 were included in the final analysis (Figure 2). The study did not reach the a priori calculated sample size due to slow accrual and difficulty in enrolling participants.

Table 1 shows the demographic and clinical characteristics of the study population at baseline—a mean of 2.7 ± 1.2 days (median, 3 days) was determined after hospitalization. The study population had a mean age of 82.9 ± 4.8 years, 54.8% were women, mean LVEF was 43.4 ± 17.4%, and 32.3% had preserved ejection fraction. The rates of hypertension, renal failure, and anemia in the study population were high. The patients had severe impairment of physical function and at least mild cognitive dysfunction at baseline.

The duration of the intervention was 8.1 ± 2.5 days in the control group and 9.1 ± 2.5 days in the EMS group, and patients in the EMS group completed a mean of 7.8 ± 1.6 EMS sessions. The mean number of steps per day of the study population was 584 ± 671 steps, and not significantly different between the two groups (*p* = 0.849). There were no adverse events during either the rehabilitation or EMS periods.

**Figure 3:**
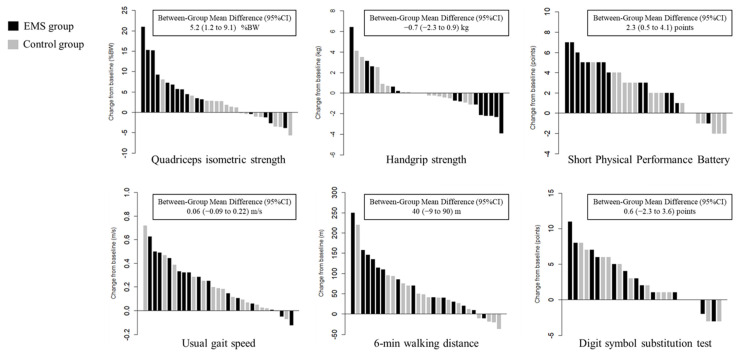
Waterfall plots of changes in physical and cognitive function. BW, body weight; CI, confidence interval; EMS, electrical muscle stimulation.

**Figure 4:**
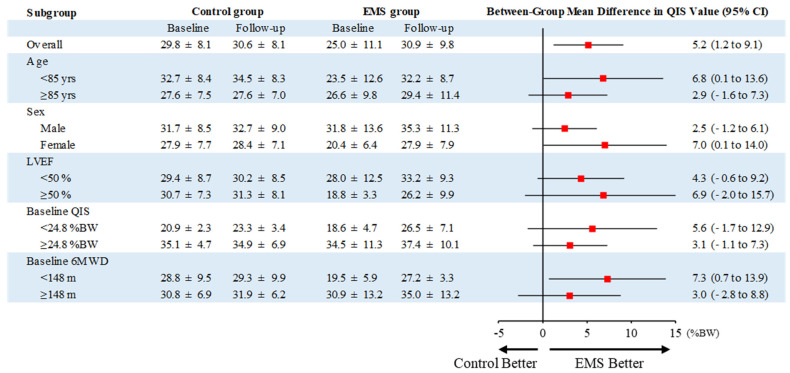
Prespecified subgroup analysis of the primary outcome. Values are expressed as means ± SD. BW, body weight; CI, confidence interval; EMS, electrical muscle stimulation; LVEF, left ventricular ejection fraction; QIS, quadriceps isometric strength; 6MWD, 6-min walking distance.

The change in QIS of the EMS group was significantly higher than that of the control group treated with early rehabilitation only (mean difference, 5.2 %BW; 95% confidence interval [CI], 1.2 to 9.1; *p* = 0.013) (Figure 3), and this effect remained uniform across the various prespecified subgroups (Figure 4). There were no significant differences in the changes in handgrip strength, usual gait speed, 6MWD, or DSST between the two groups (Figure 3). However, the change in SPPB of the EMS group was significantly higher than that of the control group (mean difference, 2.3; 95% CI, 0.5 to 4.1; *p* = 0.013), which remained uniform across the various prespecified subgroups, except in patients with preserved ejection fraction and long baseline 6MWD (Figure S1).

There were no significant differences between the EMS and control groups in change in body weight (−1.4 ± 3.2 kg vs. −1.4 ± 1.9 kg, respectively; *p* = 0.962) or BNP (−477 pg/mL, IQR −554 to −71 pg/mL vs. −502 pg/mL, IQR −654 to −135 pg/mL, respectively; *p* = 0.568). The change in QIS was significantly correlated with the changes in 6MWD (r = 0.606, *p* < 0.001), usual gait speed (r = 0.404, *p* = 0.024), and SPPB score (r = 0.547, *p* = 0.001) (Table 2). The change in body weight was significantly correlated with the change in 6MWD (r = −0.511, *p* = 0.004), but not with changes in QIS and SPPB score.

There were no significant differences between the EMS and control groups in the incidence of acute kidney injury (6.7% vs. 12.5%, respectively; *p* = 1.000) and worsening renal function (40.0% vs. 25.0%, respectively; *p* = 0.458). There were no incidences of mortality during the 90-day follow-up period. Eight patients were readmitted to hospital due to HF during the 90-day follow-up period, but there was no significant difference in the rate of hospital readmission due to HF between the EMS and control groups (26.7% vs. 25.0%, respectively; *p* = 1.000).

## 4. Discussion

The ACTIVE-EMS trial was designed to evaluate the clinical efficacy of add-on EMS therapy to exercise-based early cardiac rehabilitation in frail patients aged ≥ 75 years with AHF. The group treated with EMS therapy added to early rehabilitation showed greater improvements in lower extremity muscle strength based on the QIS than the control group undergoing exercise-based rehabilitation only, and the effects on the primary outcome remained consistent across all prespecified subgroups based on age, sex, LVEF, baseline QIS, and baseline 6MWD. As a measure of lower extremity function, analyses of SPPB score also suggested that add-on EMS therapy had clinical benefits. In this study, the EMS intervention was not associated with HF rehospitalization, although there were no adverse events during the study period. This is the first interventional trial suggesting the efficacy of EMS therapy initiated in the early period of hospitalization for AHF in frail older patients.

The results of the ACTIVE-EMS trial with regard to the effects of EMS therapy in AHF patients were consistent with previous studies showing that short-term (~2 weeks) EMS therapy in hospital improved physical function in patients with HF [9,10]. However, the patients in these previous trials were younger (around 50 years) and they had less severe impairment of physical function (mean baseline 6MWD ~240 m and 310 m, respectively) compared to the present study. In addition, the mean QIS of the patients in the present study was below the cutoff for mortality (36.2 %BW) in older HF patients [26], and their level of physical activity was very low. The results presented here indicated that EMS therapy has clinically beneficial effects on lower extremity muscle strength and function in frail older patients with AHF, and we showed that these favorable effects were consistently seen across various subgroups divided according to age (stratified at 85 years), sex, LVEF (stratified at 50%), baseline QIS (stratified at the median value), and baseline 6MWD (stratified at the median value). The EMS intervention addressed several components of frailty, including skeletal muscle weakness due to the systemic effects of aging, comorbidities, and hospital-related physical inactivity, as well as the clinical syndrome of HF and acute illness. The change in SPPB of the EMS group was significantly higher than that of the control group, but the effect was not observed in patients with preserved ejection fraction and long baseline 6MWD. Although a previous study reported that EMS might have a greater beneficial effect on physical function in severe symptomatic HF patients [29], the smaller number of patients in subgroup analysis may have reduced the statistical power to detect such an effect among these patients. Further studies regarding this issue are required in larger cohorts.

Muscle weakness in the lower extremities has been shown to be related to reductions in mobility and exercise capacity as well as poor prognosis [25,26], and is a more clinically relevant factor for the prediction of mobility disability and physical inactivity than handgrip strength [30,31]. The primary outcome of the ACTIVE-EMS trial was lower extremity muscle strength due to its marked impairment in frail older patients with AHF [16]. SPPB is a standardized, reproducible measure of global lower extremity function, which has been validated in frail, older subjects and can be used to predict a wide range of clinical outcomes, and it has also been recommended for use as a functional outcome in clinical trials in frail older subjects [32]. The present study showed that EMS improved these two key indicators, so its addition to early rehabilitation programs would be appropriate for vulnerable populations. However, EMS therapy did not improve exercise capacity as assessed by the 6MWD in the present study, in contrast to previous reports [9,10]. In this study, the body weight reduction was significantly correlated with the increase in 6MWD, but not with changes in QIS and SPPB score. Greater weight loss during hospitalization was reported previously to be correlated with increased urine output and relief of dyspnea in AHF patients [33], both of which are traditional primary goals of treatment. Taken together with these observations, the results of the present study suggest that the effects of EMS on exercise capacity in frail older AHF patients may be attenuated by the degree of recovery from AHF (i.e., those with greater weight loss during the study period), although the statistical power may have been insufficient due to small sample size.

There are some limitations of the ACTIVE-EMS trial. First, although the changes observed in this study were statistically significant, we did not reach the calculated sample size necessary to detect possible significant effects of the intervention on the primary outcome. Second, for practical reasons, the outcome assessors were not blinded to patient group allocation, which may have introduced bias favoring EMS therapy. However, the results were still likely to be valid as the assessors were trained to use a standardized technique for outcome assessment. Third, although this trial focused on short-term responses, the results did not confirm beneficial effects of the intervention on clinical events. In particular, because this study was conducted in Japan where length of stay is typically much longer than in the USA or Europe, any findings related to intervention duration or readmission may not be generalizable to other countries. In addition, the number of interventions differed among patients in this study. Further studies are needed to determine the optimal frequency and duration of EMS therapy to improve physical function and prognosis in frail older patients with AHF.

## 5. Conclusions

The inclusion of EMS therapy in addition to exercise-based early cardiac rehabilitation yielded significantly improved lower extremity function without adverse events in frail patients aged ≥ 75 years hospitalized for AHF. Short-term EMS therapy in this population showed a neutral effect on prognosis.

## Figures and Tables

**Table 1:** Baseline Characteristics.

		Control Group	EMS Group
		(*n* = 16)	(*n* = 15)
Age, years		83.3 ± 5.5	82.5 ± 4.0
	≥85	9 (56.2)	7 (46.7)
Male		8 (50.0)	6 (40.0)
Height, cm		156.1 ± 11.1	159.2 ± 10.6
Body weight at admission, kg		56.6 ± 13.7	54.4 ± 9.9
Body mass index, kg/m^2^		23.1 ± 4.4	21.6 ± 3.7
NYHA III/IV at admission, %		14 (87.5)	12 (80.0)
Systolic blood pressure, mm Hg		112 ± 20	111 ± 21
Diastolic blood pressure, mm Hg		58 ± 12	57 ± 11
Heart rate, beats/min		70 ± 12	82 ± 11
LVEF, %		43.2 ± 15.4	43.6 ± 19.9
	≥50	5 (31.2)	5 (33.3)
Diabetes, %		3 (18.8)	4 (26.7)
Hypertension, %		8 (50.0)	10 (66.7)
Dyslipidemia, %		5 (31.2)	6 (40.0)
Current smoker, %		7 (31.2)	3 (20.0)
COPD, %		2 (12.5)	2 (13.3)
Atrial fibrillation, %		5 (31.2)	6 (40.0)
Chronic renal failure, %		11 (68.8)	12 (80.0)
Anemia, %		11 (68.8)	6 (40.0)
Prior HF admission, %		5 (31.2)	3 (20.0)
Charlson comorbidity index, points		1.9 ± 1.0	2.5 ± 1.4
Laboratory data at admission			
	BNP, pg/mL	1048 [616, 1480]	664 [296, 1033]
	Albumin, g/dL	3.4 ± 0.5	3.5 ± 0.5
	Hemoglobin, g/dL	11.4 ± 2.1	12.0 ± 2.2
	Creatinine, mg/dL	1.3 ± 0.6	1.2 ± 0.4
	eGFR, mL/min/1.73 m^2^	45.8 ± 19.5	44.0 ± 21.0
Geriatric assessments			
	Mini-Cog, points	3.4 ± 1.4	2.9 ± 1.3
	SF-36 PF, points	54 ± 30	56 ± 21
	Frailty score, points	2.5 ± 1.1	2.0 ± 1.1
	SARC-F, points	3.5 ± 2.2	3.5 ± 1.7
Physical function			
	Maximal QIS, %BW	29.8 ± 8.1	25.0 ± 11.1
	Handgrip strength, kg	20.1 ± 6.9	18.3 ± 5.6
	SPPB, points	7.6 ± 1.5	5.9 ± 1.9
	Usual gait speed, m/s	0.53 ± 0.13	0.48 ± 0.16
	6-min walking distance, m	173 ± 81	155 ± 90
DSST, points		26.8 ± 9.7	20.1 ± 8.5

Values are expressed as means ± SD, *n* (%), or median [interquartile range]. BNP, B-type natriuretic peptide; BW, body weight; CI, confidence interval; COPD, chronic obstructive pulmonary disease; DSST, digit symbol substitution test; eGFR, estimated glomerular filtration rate; EMS, electrical muscle stimulation; LVEF, left ventricular ejection fraction; HF, heart failure; NYHA, New York Heart Association; QIS, quadriceps isometric strength; SF-36 PF, 36-Item Short-Form Health Survey physical functioning; SPPB, short physical performance battery.

**Table 2:** Association between the changes in cardiovascular and functional parameters.

	Δ 6MWD	Δ Gait Speed	Δ SPPB	Δ Handgrip Strength	Δ BNP	Δ Body Weight	Δ QIS
Δ QIS	0.606 *	0.404 *	0.547 *	0.069	0.035	−0.352	1
Δ Body weight	−0.511 *	−0.045	−0.265	−0.041	−0.237	1	
Δ BNP	0.266	0.293	−0.029	0.057	1		
Δ Handgrip strength	−0.035	−0.021	−0.042	1			
Δ SPPB	0.521 *	0.585 *	1				
Δ Gait speed	0.558 *	1					
Δ 6MWD	1						

BNP, B-type natriuretic peptide; QIS, quadriceps isometric strength; SPPB, short physical performance battery; 6MWD, 6-min walking distance. * *p* < 0.05.

## Data Availability

The datasets analyzed during the current study are not publicly available due to contracts with the hospitals providing data to the database.

## Supplementary Materials

The following supporting information can be downloaded at: https://www.mdpi.com/article/10.3390/jcdd9040099/s1, Table S1: Inclusion or exclusion criteria; Figure S1: Prespecified subgroup analysis of the SPPB.
