# Supplementary material for: Efficacy and Safety of Acute Phase Intensive Electrical Muscle Stimulation in Frail Older Patients with Acute Heart Failure: Results from the ACTIVE-EMS Trial
Source: J Cardiovasc Dev Dis. 2022 Mar 27;9(4):99. doi: 10.3390/jcdd9040099 (PMC9032621; doi:10.3390/jcdd9040099)
Supplement: Supplementary file 1 [file jcdd-09-00099-s001.zip › jcdd-1614791-supplementary.pdf]

Table S1. Inclusion or exclusion criteria.

Inclusion criteria

1) Patients who were hospitalized for acute exacerbation of heart failure
2) Aged 75 years or older
3) Frail patients (Short Physical Performance Battery score 4-9)

Exclusion criteria

1) B-type natriuretic peptide < 100 pg/ml at admission
2) Cognitive dysfunction
3) Acute coronary syndrome
4) Significant myocardial ischemia during low-intensity exercise
5) Resting heart rate > 120/min
6) Acute infective endocarditis, myocarditis, and pericaritis
7) Presence of a recent hospitalization for heart failure (< 1 month)
8) Severe symptomatic aortic stenosis, mitral stenosis, and hypertrophic obstructive cardiomyopathy
9) Severe pulmonary hypertension
10) Intracardiac thrombus
11) Untreated life-threatening arrhythmia
12) Resting blood pressure > 180 mmHg
13) Ongoing orthopnea
14) Requiring high inotropic support
15) Patients with circulatory assist device
16) Patients with inadequate oxygenation
17) Patients with mechanical ventilation
18) History of aortic dissection, or presence of aortic aneurysm or aortic dissection
19) Patients with uncontrolled diabetes
20) Peripheral arterial disease (Fontain III or IV)
21) Recent embolism
22) Patients undergoing hemodialysis
23) Serum creatinine > 3.0 mg/dl
24) Severe anemia
25) Short life expectancy due to advanced disease other than heart failure
26) Patients who needed assistance for walking a month before hospitalization
27) Pregnant, lactating women, patients may be pregnant

Subgroup	Control group		EMS group		Between-Group Mean Difference in SPPB score (95% CI)
	Baseline	Follow-up	Baseline	Follow-up	
Overall	7.6 ± 1.5	8.9 ± 2.9	5.9 ± 1.9	9.5 ± 1.9	2.3 (0.5 to 4.1)
Age					
<85 yrs	8.0 ± 1.0	10.1 ± 2.5	5.9 ± 2.1	9.3 ± 1.7	1.2 (-1.7 to 4.1)
≥85 yrs	7.2 ± 1.7	7.9 ± 2.9	6.0 ± 1.7	9.9 ± 2.3	3.2 (0.8 to 5.6)
Sex					
Male	8.1 ± 1.0	8.5 ± 3.0	7.5 ± 1.9	9.5 ± 2.4	1.6 (-1.2 to 4.5)
Female	7.0 ± 1.7	9.3 ± 3.0	4.9 ± 0.9	9.6 ± 1.7	2.4 (0.4 to 4.4)
LVEF					
<50 %	7.3 ± 1.6	7.9 ± 3.0	5.9 ± 1.9	9.8 ± 2.1	3.3 (1.2 to 5.3)
≥50 %	8.2 ± 0.8	11.0 ± 1.4	6.0 ± 2.0	9.0 ± 1.6	0.2 (-3.5 to 3.9)
Baseline QIS					
<24.8 %BW	7.0 ± 1.7	7.2 ± 2.9	5.8 ± 1.8	9.2 ± 2.0	3.3 (0.17 to 6.4)
≥24.8 %BW	7.9 ± 1.3	9.9 ± 2.5	6.2 ± 2.1	10.0 ± 1.9	1.8 (-0.4 to 4.1)
Baseline 6MWD					
<148 m	6.8 ± 1.6	7.4 ± 3.3	5.0 ± 1.4	9.7 ± 1.7	4.1 (1.3 to 6.9)
≥148 m	8.4 ± 0.7	10.4 ± 1.4	7.1 ± 1.7	9.9 ± 1.9	0.7 (-1.7 to 3.1)

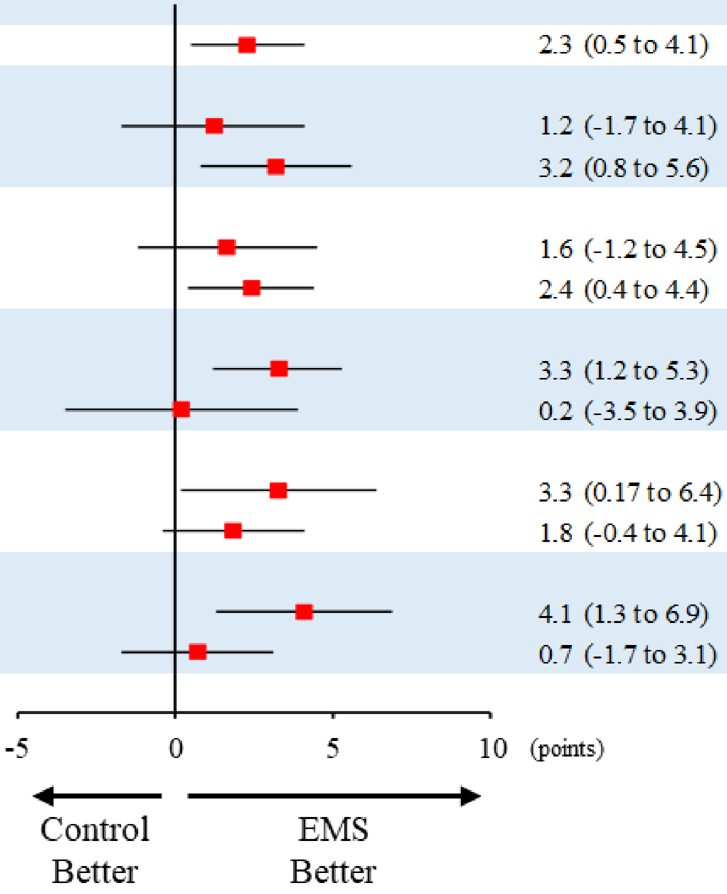

Figure S1. Prespecified subgroup analysis of the SPPB.

Values are expressed as means ± SD. BW, body weight; CI, confidence interval; EMS, electrical muscle stimulation; LVEF, left ventricular ejection fraction; QIS, quadriceps isometric strength; SPPB, short physical performance battery; 6MWD, 6-minute walking distance.
